# Sensory Attributes and Chemical Composition: The Case of Three Monofloral Honey Types from Algeria

**DOI:** 10.3390/foods13152421

**Published:** 2024-07-31

**Authors:** Rifka Nakib, Asma Ghorab, Sonia Harbane, Yasmine Saker, Akli Ouelhadj, María Shantal Rodríguez-Flores, María Carmen Seijo, Olga Escuredo

**Affiliations:** 1 Department of Plant Biology and Soil Sciences, Faculty of Science, University of Vigo, 32004 Ourense, Spain; nakib.rifka@gmail.com (R.N.); asma.ghorab@uvigo.gal (A.G.); mariasharodriguez@uvigo.es (M.S.R.-F.); mcoello@uvigo.es (M.C.S.); 2 Ecology, Biotechnology and Health Laboratory, University of Mouloud Mammeri, Tizi-Ouzou 15000, Algeria; harbane.sonia@fsbsa.ummto.dz (S.H.); saker.yasmine@fsbsa.ummto.dz (Y.S.); akli.ouelhadj@ummto.dz (A.O.)

**Keywords:** *Atractylis serratuloides*, *Retama sphaerocarpa*, *Eruca sativa*, crystallization, organoleptic characteristics, statistical analysis

## Abstract

There is a demand from the scientific, beekeeping and consumer sectors to characterize honey based on its botanical origin, as it provides unique and distinctive properties. Nevertheless, existing studies on the physicochemical properties and the sensorial profile of honey in relation to botanical origin remain insufficient. This study aimed to understand the relationships between sensory profile and various chemical compounds (minerals, sugars, water content and volatiles) of three monofloral honeys (*Atractylis serratuloides*, *Retama sphaerocarpa* and *Eruca sativa*) produced in Algeria using principal component analysis. Crystallization was detected as a distinctive attribute of *Eruca* and *Atractylis* honey. A candy aroma and odor with floral nuances, light color, crystallized state and the volatile compounds Alpha-Bisabolol and Beta-eudesmol characterized the *Atractylis* honey. *Eruca* honeys were distinguished by an animal and degraded odor, bitter taste, light color and the presence of Dimethyl trisulfide and Dimethyl tetrasulfide. Finally, a vegetal aroma, some saltiness and sourness, dark amber color, lower sugar content, higher K content and Lilac aldehyde and Lilac aldehyde D characterized *Retama* honeys.

## 1. Introduction

Research on honey is crucial due to the documented medical, antioxidant and antibacterial capacities of this bee product [1,2]. In addition to the acquired interest as an agri-food product, the cosmetic industry is also focused on honey due to the beneficial qualities reported. These properties are attributed to the botanical and geographical origin, which determine differences among honeys and influence market prices. Therefore, different economic sectors (beekeepers, public entities, consumers, among others) demand accurate characterizations of honey. Moreover, the quality and a confirmed authenticity are factors that influence the purchasing decisions of honey consumers [3,4]. Some reported insights with direct effects on honey price are sensorial perceptions (color, taste and odor), crystallization state, packaging and best-before date [4,5].

The most desired honey types are those from a single botanical source (monofloral honey) because the honey bees forage in specific plant species, enabling harvesting of some chemical compounds that can provide some interesting healthy properties. Experimenting with less known or less researched honey types is a recently established goal. In Algeria, the most studied monofloral honey types are *Ziziphus*, *Eucalyptus*, *Euphorbia*, *Citrus* and *Hedysarum* [6,7,8,9,10]. *Retama* honey, *Atractylis* honey and *Eruca* honey are less known, but recent research on their botanical profile, antioxidant activity and volatile compounds makes them especially attractive for local and international trade [11,12]. Since honey is a substance produced by honey bees primarily from the nectar of flowers or secretions from living parts of plants, it is important to investigate the benefits of its botanical source. *Retama* honey derived from *Retama* species (Fabaceae family), mainly *R. sphaerocarpa* shrub, is a shrub widely represented in the Mediterranean region of northeastern Africa, especially in the dry desert of Algeria and the Iberian Peninsula [13]. There are studies that highlight some properties derived from extracts of *Retama sphaerocarpa* and *R. raetam,* as is the case of high antibacterial activity [14]. The *Atractylis* honey type is obtained mainly from the species *Atractylis serratuloides* (Asteraceae family), which is a thorny shrub growing in dry steppes. The plant is well-known in Mediterranean folk medicine for its diuretic effects and applications against circulatory disorders [15]. In the case of *Eruca sativa* (Brassicaceae family), it is an annual, upright plant with a strong fragrance when it flowers. Phytochemicals extracted from *E. sativa* have been shown to be advantageous and health-promoting compounds for human diets [16].

Sensory analysis is the scientific method of assessing the organoleptic properties of foods and plays an important role in establishing the authenticity of products [17,18]. This method of examination involves the basic human senses, participating in the acceptance and evaluation of honey as a food product. Although sensory analysis is dependent on human appreciation, it is a useful tool and in some foods it becomes irreplaceable in the assessment of quality [4,17,19,20]. Color, aroma, taste, texture and visual perception in addition to the viscosity of honey are key factors in determining honey quality and in the sensory acceptability [19,21]. The color is the first visual attribute that the consumer perceives to establish a link with beneficial properties of honey. The variety of honey colors is well known, ranging from pale white to dark hues [22]. Some researchers showed the influence of certain chemical compounds on the color of honey, in particular polyphenols, carotenoids and minerals [3,23,24,25]. Although there is limited research on the sensory profile of Algerian honeys [6], the close relationship between sensory attributes, some chemical compounds in honey, and the botanical origin have been reported [20,21,23,26]. Furthermore, honey is a viscous solution with high monosaccharides content and is often susceptible to crystallization. The state of crystallization depends on the water content, the presence of nucleation seeds, the degree of supersaturation and the viscosity, which in turn are related to temperature [21]. Many consumers, including Algerians, are accustomed to viewing crystallized honeys as altered or adulterated products. However, this is false for several scientific reasons. Crystallization is a common natural phenomenon that can also indicate the authenticity of honey and, in most cases, has no effect on nutritional value. Some honey types crystallize naturally and correctly, just after honey extraction [27]. However, many beekeepers in Algeria report this phenomenon and express their concern about the poor marketing of these honey types. A correct form of crystallization is a desired phenomenon for the production of creamy and spreadable honeys that are recently becoming popular for some consumers. Otherwise, improper crystallization can increase water activity, which can then cause microbial fermentation [28].

Despite the rich biodiversity of the Algerian landscape and the wide range of honey varieties that can be produced, the population exhibits a low consumption of honey, primarily from a limited number of types known for their biological benefits [11,22]. Among the least sought-after and yet uncharacterized honeys, whether in Algeria or worldwide, are honeys from the previously cited botanical sources: *Retama sphaerocarpa* (Retem honey), *Atractylis serratuloides* (Sor honey) and *Eruca sativa* (Harra honey). These honeys have recently been characterized for the first time [11,12], supporting their first presentation to the local consumer and also to the scientific community as interesting honey types with potential applications in multiples aspects. In this study, chemometric approaches were employed to assess the relationships among the physicochemical profile, mineral and sugar content, specific volatile compounds, crystallization ratios and sensory attributes of samples from these three honey types. The expert assessors established basic sensory characteristics to confirm the impact of the mentioned compounds on the specific sensorial attributes of the honey samples.

## 2. Materials and Methods

### 2.1. Geographical and Botanical Origin of Honey Samples

**Figure 1:**
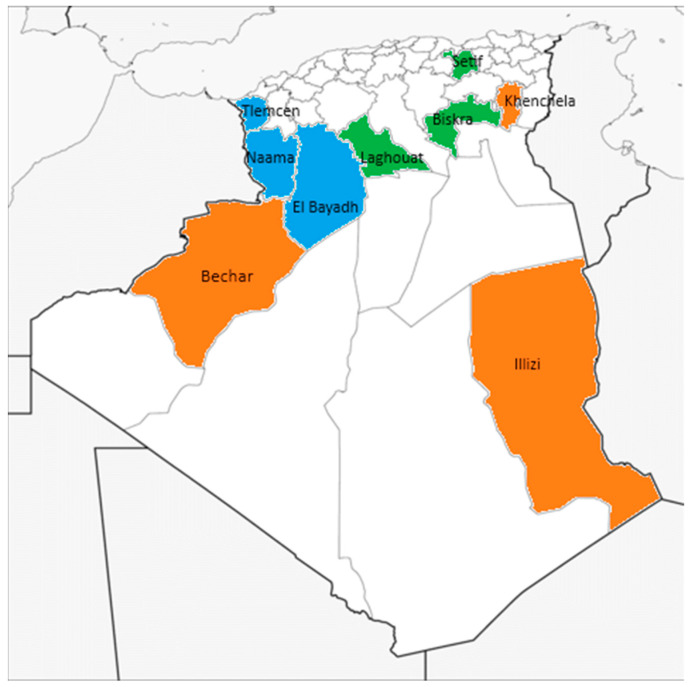
Geographical location of the honey samples: *Atractylis* honeys (shaded in blue), *Eruca* honeys (orange) and *Retama* honeys (green).

Twenty-two monofloral honeys of three different types collected in arid and semi-arid regions of eastern and western Algeria were analyzed. Specifically, six samples of *Retama sphaerocarpa* honey, eleven of *Atractylis serratuloides* honey and five of *Eruca sativa* honey were selected for the present analyses. *Atractylis* samples were taken from Naama, Tlemcen and El Bayadh; *Eruca* samples from Khenchela, Bechar and Illizi; and the *Retama* samples from Biskra, Setif and Laghouat, as shown on the map in Figure 1. The samples were collected directly from beekeepers over a spring period. The pollen spectra of the samples were already determined by melissopalynological method, recently published by Nakib et al. [12]. In *Atractylis* honey samples, the *Atractylis serratuloides* pollen type had a mean representation of 32.0% in the pollen spectrum. In the group of *Eruca* honey, a mean value of 72.4% was calculated for the *Eruca sativa* pollen, and in the case of *Retama sphaerocarpa* honey, the pollen type *Retama* was represented with a mean value of 71.4%. Additionally, routine quality parameters such as Pfund color, water content and electrical conductivity were determined in the study [29].

### 2.2. Determination of Sensory Attributes: Descriptors and Assessors

The sensory analyses of the honey samples were carried out by a group of five trained sensory assessors, previously selected and trained according to the ISO 8586:2023, Sensory analysis: Selection and training of sensory assessors [30]. A suitable and odorless room was set up, under natural light and room temperature. The honey samples were presented to the panelists in 20 mL quantities in small transparent glasses; water was provided to rinse the mouth between sample tastings. They evaluated the honey samples of different origins for their global characteristics (visual, olfactory and gustatory) on a scale from 0 to 10 for each global attribute. In the case of color, the scale went from the clearest (0) to the darkest notes (10). The olfactory perceptions (odor and aroma) and gustatory attributes (basic taste) were evaluated using a scale for the intensity (from lowest to highest) marking the correspondent descriptors. The parameter of state was evaluated numerically using a predefined scale. Liquid samples were assigned a score of 1, samples in the process of crystallization or that were creamy were given a score of 5, and fully crystallized samples were assigned a score of 10. The spice level and astringency were evaluated based on presence or absence. Table 1 shows a summary of the descriptors used in the honey tasting sheet.

### 2.3. Determination of Mineral Content

For the quantitative determination of mineral content, the first step involved an extraction from honey samples carried out using microwave digestion according to the methodology proposed by Caroli et al. [31]. The physical condition of the honey samples required prior homogenization before analysis. Thus, the samples were quietly heated to approximately 50 °C and dissolved by ultrasonic agitation. Aliquots of 0.5 g of honey were taken and transferred in a CEM MARSX model microwave oven, where they were subjected to digestion. In these, 9 mL of nitric acid and 2 mL of hydrogen peroxide were already added. Finally, the residue was made up to 25 mL with distilled water and Mg, Cu, Ca, Fe, P and Zn were quantified by atomic absorption (Varian SpectrAA-220 Fast Sequential), while Na and K were quantified by atomic emission. The standards for each mineral element were prepared from 1000 mg/mL of stock solutions in 10% nitric acid by dilution with high-purity deionized water. The results were expressed in mg/kg.

### 2.4. Determination of Sugar Composition

The sugars (fructose, glucose, saccharose, maltose, turanose and melecitose) were determined by high-performance anion exchange chromatography (HPAEC) using a Dionex ICS-3000 ion SP chromatograph (Sunnyvale, CA, USA) [27]. First, honey sample solutions (10 mg/L) were prepared in milli-Q water and filtered through a 0.45 μm diameter pore filter to eliminate any potential impurities or large pollen grains. Honey solutions (10 µL) were injected into the loop of the chromatograph. A CarboPac PA1 column (3 × 250 mm) (polyvinylidene/polyvinylbenzene column suitable for mono-, di-, tri- and oligosaccharide analysis) was used to separate the sugars. Sugars were detected using a gradient of two mobile phases (A and B) in a pulsed amperometric detector (PAD). Phase A was ultrapure water, while phase B was 200 mM NaOH (HPLC grade, Merck, Darmstadt, Germany). For every pure sugar (Sigma-Aldrich, Madrid, Spain), standard solution calibration curves were made to determine the amount of sugar present in the honey samples. The linear ranges of identified sugars were between 10 and 45 mg/L (glucose and fructose), 0.5 and 10 mg/L (sucrose, maltose) 0.1 and 10 mg/L (turanose, melezitose). The Chromeleon 7.3. Chromatography Management System software was used for the acquisition of all chromatograms.

### 2.5. Determination of Volatile Compounds

Volatile compound extraction in honey samples was performed by the headspace solid-phase microextraction (HS-SPME) method. Gas chromatography–mass spectrometry (GC-MS) analysis was used for the separation of the compounds with a capillary column ZB-5MSi column. The methodology and conditions of the extraction are detailed by Nakib et al. [12].

### 2.6. Statistical Analysis

The obtained sensory and physicochemical data were compared using ANOVA and the post hoc Bonferroni test to compare the means between the three honey types (*p* < 0.05). After that, a principal component analysis (PCA) was applied to examine the relationships between the main physicochemical parameters and sensory data, as well as to explore the distribution of honey samples based on botanical origin. This analysis reduces dimensionality and extracts the most relevant information from samples, helping to understand relationships between parameters and groups of samples. The results are shown graphically in a biplot, extracting information from the components that represents a greater percentage of relevant information. Statistical analyses were carried out using Statgraphics Centurion V18 software for Windows (Statgraphics Technologies, Inc., The Plains, Fauquier County, VA, USA) and SPSS Statistics 29 for Windows (IBM, Somers, NY, USA).

## 3. Results

### 3.1. Sensory Profile of Honeys

**Figure 2:**
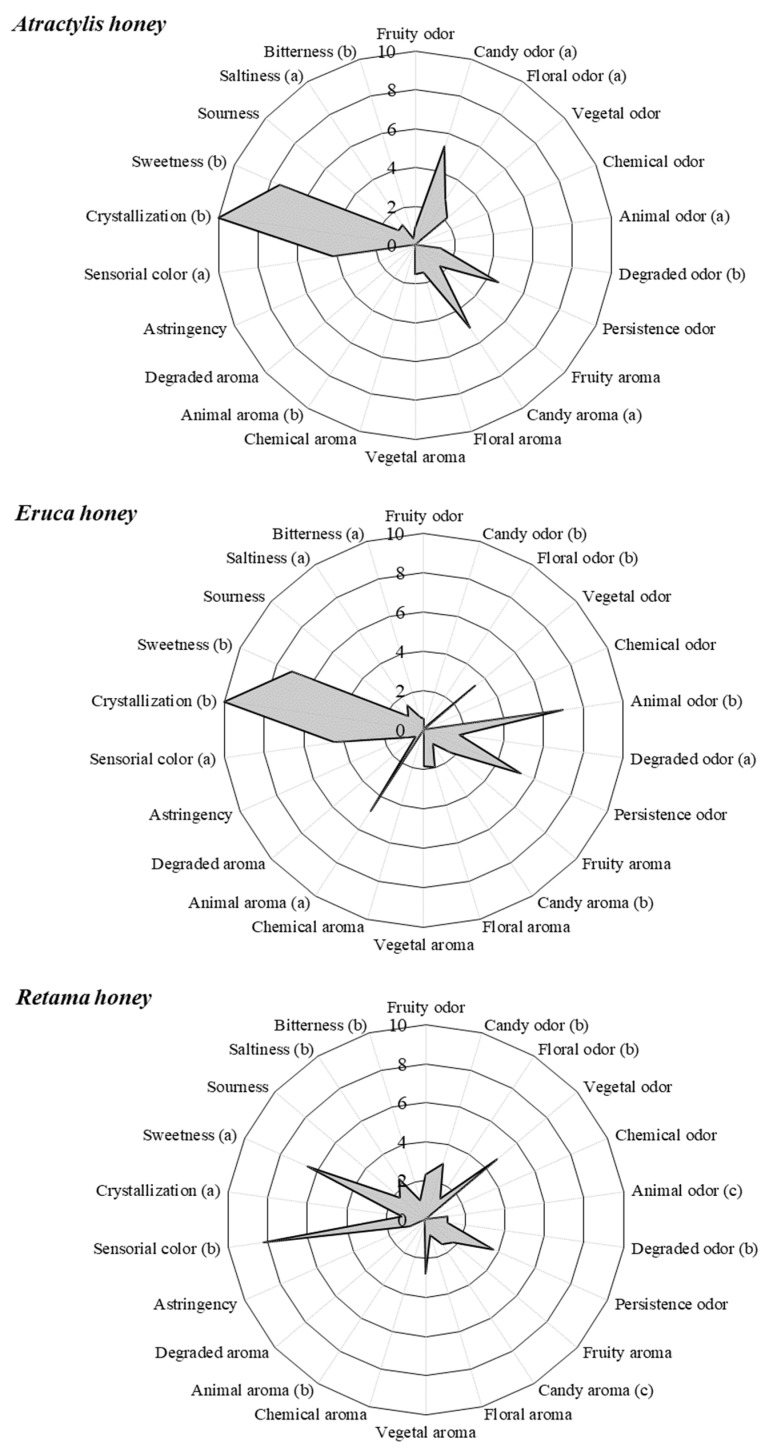
Graphical representation of the sensorial profile of each honey type. Different letters indicate significant differences between the mean data by honey type according to Bonferroni test (*p* < 0.05).

The honeys investigated were divided into three different profiles according to botanical origin. The physiological state (crystallization), the degree of color perceived visually, the basic taste, and the various odor and aroma descriptors (sensations perceived by the olfactory organ through the retronasal route) are represented in Figure 2. The attributes, statistically significantly different by type of honey (indicated with letters in parentheses in Figure 2; *p* < 0.05), marked the distinctive sensory profile. Thus, Atractylis honey samples were characterized homogeneously by the assessors as crystallized honeys, with a light color and a candy vanilla/floral odor and also candy vanilla aroma. Samples of this type were also scored as extremely sweet due to their basic taste. The samples from Eruca honey were also in a crystallized state, presenting a light color (between extra-light amber and light amber), along with a persistent animal odor and often vegetal traces, coinciding with the aroma. The Retama honey samples presented an amber dark color and accentuated candy and vegetal. The aroma was mainly vegetal with low intensity, although some animal and degraded nuances were also detected, and the taste was sweet with salty traces. The assessors noted some samples were in progress of crystallization. The sweet taste of this honey type was the least detected in comparison to the other types.

### 3.2. Physicochemical Parameters of Honey Samples

#### 3.2.1. Mineral Content by Honey Type

The mineral elements quantified in the studied samples were K, P, Ca, Na and Mg, but Cu, Fe and Zn were identified in very low concentrations (Table 2). K is the most abundant mineral, with a mean value of 1041.8 mg/kg, but it is significantly higher in Retama samples, compared to the other two honey types (*p* < 0.05). P was the second most abundant mineral in the honey samples with an average of 361.1 mg/kg, with Retama samples having a significantly higher content compared to Atractylis and Eruca samples (*p* < 0.05). The mineral elements Ca, Na and Mg represented average values below 100 mg/kg in all the honey types. Mg was the lowest one (33.6 mg/kg), but it was considerably higher in Retama honey (*p* < 0.05). The samples of this honey type also had significantly higher values of Ca (*p* < 0.05) and slightly lower levels of Na, although this was not statistically different. On the contrary, Atractylis honey and Eruca honey had higher average values of Na (48.0 mg/kg and 46.2 mg/kg, respectively).

#### 3.2.2. Sugar Content and Sugar Ratios in Honey Samples

In the analyzed samples, six sugars were identified and quantified: fructose, glucose, saccharose, melezitose, turanose, and maltose. The average fructose content and glucose content in the honey samples were 39.2% and 32.3%, respectively (Table 3). The average fructose content was similar between the three honey types but slightly higher in the *Atractylis* samples. However, *Retama* samples had a significantly lower average glucose value (27.2%) compared to *Atractylis* and *Eruca* samples (33.3% and 36.0%, respectively, *p* < 0.05). Turanose was the third most abundant sugar, with a mean content of 3.2%, and a significantly higher average content in *Retama* samples (3.6%, *p* < 0.05). Maltose was the next most relevant sugar with an overall average content of 1.1%. *Atractylis* and *Retama* honeys had significantly higher mean maltose concentrations, with 1.4% and 1.0%, respectively, compared to *Eruca* honeys (0.4%, *p* < 0.05). Saccharose was detected in all the samples, with an average concentration of 0.3%, but it was significantly higher in samples from *Atractylis* and *Retama* (0.4% and 0.2%, respectively). Finally, melezitose was identified at low concentrations (mean value of 0.1%) and was significantly higher in *Eruca* and *Retama* honeys (0.07% and 0.08%, respectively; *p* < 0.05).

Water content is an important physicochemical parameter for the evaluation of honey quality but also due to its influence on the crystallization process. The average water content in the analyzed honey samples was 15.2%, significantly higher in *Eruca* samples (19.1%) than the other honey types (*p* < 0.05). The sum of fructose and glucose (F+G) was represented by significantly higher mean values for the *Atractylis* and *Eruca* samples (73.4% and 74.4%, respectively), compared to the Retama samples (65.5%). The average value of F/G ratio was significantly different between the three honey types, finding the highest value in *Retama* honey (1.4). Regarding the G/W ratio, *Atractylis* honeys had a value of 2.3, which was significantly higher than *Retama* and *Eruca* honeys, with equal average values of 1.9. Finally, *Retama* samples were the darkest and those with the highest electrical conductivity (average values of 96, according to the Pfund scale, and 628.7 µS/cm, respectively), while the other two types of honey had a similar color (around 50) and electrical conductivity (around 300 µS/cm).

#### 3.2.3. Main Volatile Compounds of Honeys

The representative volatile compounds identified in each type of honey are summarized in Table 4. Beta-eudesmol and Alpha-bisabolol are volatile compounds exclusive to *Atractylis* honey samples. *Eruca* samples are characterized by Dimethyl trisulphide and Dimethyl tetrasulphide as representative volatile compounds, to which Acetophenone and Benzeneacetic acid ethylester are added. Regarding *Retama* samples, Lilac aldehyde and Lilac aldehyd D were the most common ones.

#### 3.2.4. Main Physicochemical Compounds and Sensory Attributes Involved in the Botanical Classification of Honey Samples

**Figure 3:**
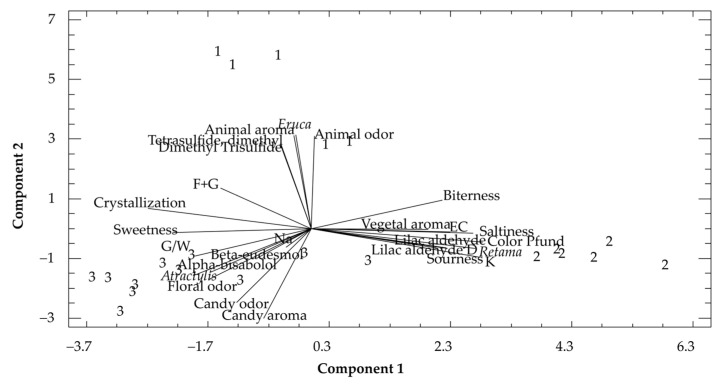
Principal component analysis of some measured parameters on the studied honeys. 1. *Eruca sativa* honeys, 2. *Retama sphaerocarpa* honeys, 3. *Atractylis serratuloides* honeys. EC: electrical conductivity; F+G: fructose + glucose; G/W: glucose/water ratio.

A principal component analysis of the physicochemical variables and sensorial attributes measured was carried out to verify the relationships between them (Figure 3). The statistical procedure extracted seven components that explained 83.3% of the variance of data. The two first components explained the highest percentage of data variance (64.1%). The variables with the highest weight in the two first components were K, color, Dimethyl trisulfide, Dimethyl tetrasulfide, candy (odor and aroma), animal (odor and aroma), saltiness, *Retama sphaerocarpa* pollen and *Eruca sativa* pollen (with a value greater than 0.28). The projection of the variables (sensorial, physicochemical and pollen) on the first two components of the PCA is presented in Figure 3. In the lower quadrant of the plot, the F+G ratio, the degraded aroma, Dimethyl tetrasulfide and Dimethyl trisulfide volatile compounds with the *Eruca* pollen type, and the honey samples from *Eruca* are situated. On the other hand, in the upper right quadrant, K, saltiness, sourness, EC (electrical conductivity), color, vegetal aroma and the volatile compounds Lilac aldehyde and Lilac aldehyde D are placed together with *Retama sphaerocarpa* pollen and the honey samples of this botanical origin. Finally, in the lower right quadrant, crystallization, sweetness, floral odor, G/W ratio, candy odor and aroma are situated positively with *Atractylis serratuloides* pollen and most *Atractylis* honey samples.

## 4. Discussion

Most of the characterized Algerian honeys are based on multifloral honeys or monofloral types from jujube, euphorbia or eucalyptus species, among others, originating mainly from the Mediterranean area of the north of the country. This study presents the characteristics of three monofloral honey types (Sor honey, Harra honey and Retem honey), relatively little known by consumers and scientists, linking organoleptic attributes to some physicochemical compounds, pollen properties and representative volatile compounds. The results showed significant differences between the analyzed physicochemical parameters and compounds by honey type. Fructose and glucose are generally the most studied monosaccharides, because they are the main compounds in honey. The sum of these sugars (F+G) is a criterion established in honey standards to evaluate the quality and origin [27]. It should be higher than 60 g/100 g for the case of blossom honey and higher than 45 g/100 g for honeydew honey [32]. However, the concentration of both sugars can be an indicator of a determined botanical origin, as had been suggested by some authors, where sugars are effective compounds to differentiate between monofloral honey types [33]. Even the presence of melezitose, despite the low content that often presents in honey, was referenced as an indicator of the presence of honeydew [34,35,36]. Different types of blossom honey generally contain the same sugars, but in significantly varied amounts [37]. Therefore, sugar composition is related to the flora and, to a lesser extent, the climate and geographical origin. The *Retama* honeys analyzed in the present study presented lower sugar concentrations compared to *Atractylis* and *Eruca* honeys. The three honey types had an average F+G content between 65.5% and 74.4% and the melezitose and turanose concentrations were low (mean value of 0.1% and 3.2%, respectively), in accordance with the quality criteria for blossom honeys. Sucrose content is also used as a quality measure for honey, and it must be less than 5 g/100 g, with exceptions for some monofloral honeys such as lavender and acacia, among others [32]. This criterion could be related to an adulteration of the product or inadequate feeding of honey bees with sugar syrups, which can be responsible for food fraud. In the case of the studied Algerian honeys, none exceeded a concentration of 5 g/100 g. The water content of honey is a parameter linked to botanical and geographical origin, climatic conditions, season, nectar humidity, and the degree of honey ripening. As honey is a highly hygroscopic product, the water content can vary during storage, leading to an increase in water in the upper layers. This parameter influences crystallization trends normally related to botanical origin and honey management; however, when the water content is high, this facilitates the fermentation of honey by yeasts [32]. Typically, honey appears in a liquid state, but most honey eventually turns to a crystallized state more or less promptly, causing the honey to change into a solid form. This crystallization state plays a fundamental role in the overall quality perceived by consumers [21]. In terms of honey management, the tendency of honey to crystallize is directly related to the water content and main sugars of honey. Therefore, some sensitive parameters such as water content, the sum of fructose and glucose (F+G) and their ratio (G/W and F/G) are used as crystallization indicators. In the case of the studied samples, *Atractylis* honey presented the highest tendency to crystallize, having a G/W ratio of 2.3.

The main resource for mineral content in honeys is the nectar of the plants. These plants take minerals from soil, so factors such as geographical origin and botanical origin are important because each type of soil is accompanied by different plant communities growing in these areas. Despite the low concentration of minerals in honey, they are very useful for the characterization of honey due to their association with geographical and botanical origins [38]. K was the main mineral detected, followed by P, Na, Ca and Mg. *Retama* honeys had a significantly higher content of K, P, Ca and Mg than the other two honey types analyzed. Similar results for some Mediterranean Algerian honey samples have been found [6]; however, lower concentrations have been found for west Algerian honey samples [39]. The Na content in honey is often higher in samples collected near the sea, because it has some influence on these contents [40] but also in honey samples collected in plants growing in salty soils, for example in arid areas, as was found in the honeys analyzed in the present study. The highest values correspond to *Atractylis* and *Eruca* samples, both from arid areas. It is well known that dark honeys have a higher mineral content [6]; this is the case of the *Retama* honey samples, the darkest of the three honey types investigated. Minerals also influence the basic taste since they are present as salts and excite the salty papillae. This perception was reflected by the assessors in the case of the samples of this honey type.

To characterize the sensory profile of the analyzed honey samples, the findings regarding the essential volatile compounds identified in a previous study have been integrated in this work [12]. In the present research, the sensory profiles of the three honey types were different and distinguished from each other. Assessors agreed on the crystallized state of all *Atractylis* honey samples during the sensory test, which could be caused by the low water content noted for this honey type and their relation to glucose content. Glucose is less water-soluble than fructose; therefore, a high glucose content favors the process, as found in *Atractylis* and *Eruca* honeys [27,36,41]. Samples of *Atractylis* honey had an average G/W ratio higher than 2.0, leading to rapid crystallization [42]. For their basic tastes, *Atractylis* honey samples were rated as sweet and had vanilla or white chocolate (candy) aromas with floral traces assumed (Figure 2). The odor attribute had judgments of candy (vanilla) with some floral contribution for this honey type also. Regarding its volatile compounds, Alpha-linalool and Beta-eudesmol were among the important volatile compounds of *Atractylis* honey, playing roles as a volatile plant component and an eudesmane sesquiterpenoid [12]. Some authors confirmed that linalool and linalool oxide were responsible for the floral odor, also contributing to the aroma of plant parts of the Adoxaceae family [43], while Beta-eudesmol stood out for its contribution to the woody fragrance of branch oil [44]. Recent identifications of volatile compounds in monofloral honeys showed the influence of specific active volatile compounds at lower concentrations on the basic taste of honey [44]. In recent decades, there has been a notable shift in consumer behavior, accompanied by a gradual increase in the consumption of healthy foods. This has led to an increased focus on the quality and nutritional value of food. The employment of SPME and other non-destructive VOC analysis techniques is particularly well-suited to fulfill these requirements because they necessitate minimal or no sample preparation and are environmentally friendly. Plants are capable of synthesizing an extensive range of primary and secondary metabolites, which exhibit a diverse array of properties and biological functions. The plethora of volatile metabolites, derived from an array of essential nutrients, including amino acids, fatty acids and carotenoids, may contribute to overall health and well-being. Some studies suggest that beta-eudesmol may be a promising candidate for further development as a drug with anticancer and antimutagenic activities [45,46]. Alpha-Bisabolol is a monocyclic sesquiterpene of notable pharmacological interest, derived naturally from a multitude of plant species, including *Atractylis gummifera* [47]. Volatile sulfur compounds are frequently potent odorants and biologically active elements [48]. Lilac aldehydes were identified as a significant contributor to the volatile profile of *Retama*, which was described as a floral aroma reminiscent of Lilac. The antibacterial activity of certain aldehyde compounds present in hive products, such as beeswax, has been the subject of scientific investigations [49].

*Eruca* honeys, visually considered crystallized honeys at room temperature, had the lowest (F/G) ratio (below of 1.1 for all samples). However, this honey type had a high average water content, which may be explained by early harvesting or perhaps by the nature of the plant itself, as *Eruca sativa* honeys have never been studied for their water content [12]. In addition, the samples appear to undergo rapid crystallization, since the G/W ratio of the highest values exceeds 2.1 [42]. It is important to mention that some types of honey crystallize naturally and correctly just after honey extraction. Frequently, beekeepers, in particular in Algeria, report this phenomenon and express their concern, as already mentioned, about the poor marketing of this honey type. The low content of certain minerals in these honey samples is one of the reasons for its light color. Its sweeter basic taste is due to the high glucose and fructose content. The assessors noted an unpleasant aroma split between vegetal, animal, and mineral, which may be caused by glucosinolates formed mainly of sulfur and nitrogen, which are responsible for the pungent basic taste and odor and were found among the volatile compounds of this honey type [12].

Samples of *Retama* honey were not noted as totally crystalized, but with a heavy viscous appearance. Based on the results, they tend to crystallize slowly at room temperature, because their G/W ratio is between 1.7 and 2. This is also confirmed by the range of F/G ratio values that are greater than 1.3, showing that these samples will either remain liquid during storage or crystallize very slowly [27,37,50]. In terms of color, the samples range from dark to dark amber, which can be explained by their mineral richness that is proportional to the blackness of the final product. Their richness in phenolic compounds (mainly flavonoids) is also a reason, as already indicated in previous research [3,12,51]. The odor was noted as candy with vegetable traces and a sweet basic taste associated with a slight saltiness. In terms of aroma, the samples were characterized by vegetal and slightly floral aromas. According to the literature, the typical caramel odor results from a complex balance between fruity, vegetal, spicy, nutty and caramel notes arising from the presence of carboxylic acids, aldehydes, oxygenated heterocyclic compounds, ketones and carbocyclic compounds [52]. This could be the reason for the odor as aldehydes including nonanal, decanal, Lilac aldehyde D and Lilac aldehyde, benzene derivatives, and ketones are the most concentrated and frequent compounds in *Retama* honey [12].

The differences in honey type attributed to the most significant sensory attributes and chemical compounds were corroborated by the PCA. *Eruca sativa* honey samples are grouped at the ratio of the two main sugars (F+G), which is quite high in this type of honey. In addition, the two volatile compounds Dimethyl tetrasulfide and Dimethyl trisulfide may explain the bitterness and degraded aroma reported by the assessors. Regarding *Retama* honey samples, the intensity of color is also explained by the high electrical conductivity and the mineral content. Honey producers should keep in mind that crystallization is a valuable parameter to influence consumer acceptance. However, information on the botanical origin is essential, and as has been demonstrated in the present study, monofloral honeys from *Atractylis* and *Eruca*, despite their crystallized state, have interesting sensory attributes for the consumer.

## 5. Conclusions

The current study used advanced statistical techniques, such as PCA, to show how certain chemical compounds (mostly derived from plants) are closely associated with the sensory properties of some Algerian monofloral honeys. Sensory analyses of *Atractylis serratuloides*, *Retama sphaerocarpa* and *Eruca sativa* monofloral honeys revealed three different sensory profiles. Furthermore, crystallization is a distinctive state attribute that is known to occur naturally in honey and can be used to verify the authenticity of honey derived from *Eruca* and *Atractylis*. Odor and aroma were connected to the volatile makeup of the current honey varieties that were previously investigated. The color intensity, mineral content and botanical provenance of the honey samples were also explained. The particular physiochemical characteristics for each type of honey were reflected in the PCA, extracting the most outstanding volatile compounds, sugars, minerals and sensory attributes. Among the most notable characteristics of *Atractylis* honey, its clear crystallized state, aroma and smell of caramel with floral nuances, and the volatile compounds Alpha-Bisabolol and Beta-eudesmol were differentiated. *Eruca* honeys were distinguished by their animal and degraded odor, bitter taste, light color and the presence of Dimethyl trisulfide and Dimethyl tetrasulfide. Retama honeys were mainly characterized by a vegetal aroma, salty and acidic flavor, dark amber color, lower sugar content, higher K content and the volatile compounds Lilac aldehyde and Lilac aldehyde D. The findings of this study offer consumers in Algeria and the beekeeping industry valuable information about the legitimacy of these poorly studied monofloral honeys. In the future, the database of the physicochemical and sensory characteristics of these honeys will be expanded to contrast these first results.

## Figures and Tables

**Table 1:** Attributes considered for the sensorial description of samples.

Texture	Color	Odor and Aroma	Basic Taste	Astringency	Spicy
Liquid Creamy/Crystallized	White Light amber Amber Dark amber Dark White Straw Gold Orange Brown	Fruity: orange, apple, other. Candy: vanilla, caramel, white chocolate, other. Floral: orange, blossom, lavender, violet, rose, other. Vegetal: fresh grass, mint, leaves, resin, wood, pepper, cinnamon, other. Chemical: lactic, other. Animal: dog, urine, leather, wax, other. Degraded: soap, smoked, burned, caramelized, other.	Sweetness Sourness Saltiness Bitterness	Yes No	Yes No

**Table 2:** Descriptive analysis (mean value ± standard deviation and range) of mineral elements (mg/kg) of studied honeys.

	*Atractylis* Honey	*Eruca* Honey	*Retama* Honey	Total
K	645.8 a ± 260.0	421.2 a ± 36.7	2284.18 b ± 274.4	1041.8 ± 815.9
	(342.0–1274.0)	(361.0–460.0)	(449.0–2608.0)	(342.0–2608.0)
P	332.6 a ± 37.1	315.6 a ± 22.6	451.2 b ± 34.2	361.1 ± 65.3
	(277.0–380.0)	(296.0–344.0)	(277.0–494.0)	(277.0–494.0)
Ca	63.2 a ± 9.2	60.8 a ± 6.5	83.7 b ± 14.1	68.2 ± 13.8
	(49.0–76.0)	(51.0–69.0)	(71.0–112.0)	(49.0–112.0)
Na	48.0 ± 14.7	46.2 ± 25.8	39.2 ± 12.4	45.2 ± 16.8
	(28.0–70.0)	(25.0–79.0)	(26.0–55.0)	(25.0–79.0)
Mg	17.7 a ± 5.5	24.4 a ± 3.0	70.5 b ± 6.2	33.6 ± 23.8
	(11.0–27.0)	(20.0–28.0)	(24.0–76.0)	(11.0–76.0)
Cu	<1.0	<1.0	<1.0	<1.0
Fe	<1.0	<1.0	<1.0	<1.0
Zn	<0.5	<0.5	<0.5	<0.5

Different letters indicate significant differences according to the Bonferroni test (*p* < 0.05) between the mean values by honey type. Total: all honey samples.

**Table 3:** Descriptive analysis (mean ± standard deviation and range) of sugars, water content, electrical conductivity and color by honey type.

	*Atractylis* Honey	*Eruca* Honey	*Retama* Honey	Total
Fructose (%)	40.1 ± 1.6	38.4 ± 1.8	38.3 ± 0.4	39.2 ± 1.6
	(37.5–42.2)	(36.6–41.2)	(37.6–38.8)	(36.6–42.2)
Glucose (%)	33.3 a ± 2.3	36.0 a ± 2.2	27.2 b ± 1.6	32.3 ± 4.0
	(29.2–37.0)	(33.7–39.3)	(24.7–29.4)	(24.7–39.3)
Turanose (%)	3.1 a ± 0.4	2.7 a ± 0.2	3.6 b ± 0.2	3.2 ± 0.5
	(2.4–3.6)	(2.3–2.9)	(2.4–3.9)	(2.3–3.9)
Maltose (%)	1.4 a ± 0.5	0.4 b ± 0.1	1.0 a ± 0.4	1.1 ± 0.6
	(0.8–2.2)	(0.2–0.5)	(0.3–1.5)	(0.2–2.2)
Saccharose (%)	0.4 a ± 0.3	0.02 b ± 0.10	0.2 a ± 0.13	0.3 ± 0.3
	(0.1–0.9)	(0.01–0.2)	(0.1–0.4)	(0.01–0.9)
Melecitose (%)	0.03 b ± 0.01	0.07 a ± 0.03	0.08 a ± 0.04	0.1 ± 0.03
	(0.02–0.04)	(0.05–0.1)	(0.01–0.1)	(0.01–0.1)
Water content (%)	13.9 a ± 1.6	19.1 b ± 2.1	14.3 a ± 1.0	15.2 ± 2.7
	(12.2–17.6)	(15.7–21.0)	(13.3–15.6)	(12.2–21.0)
F+G	73.4 a ± 2.0	74.4 a ± 3.8	65.5 b ± 1.7	71.4 ± 4.5
	(71.0–77.4)	(71.1–80.5)	(63.0–67.8)	(63.0–80.5)
F/G	1.2 a ± 0.1	1.1 b ± 0.01	1.4 c ± 0.1	1.2 ± 0.2
	(1.1–1.4)	(1.0–1.1)	(1.3–1.6)	(1.0–1.6)
G/W	2.3 b ± 0.3	1.9 a ± 0.3	1.9 a ± 0.1	2.1 ± 0.3
	(1.8–2.6)	(1.6–2.3)	(1.7–2.0)	(1.6–2.6)
EC (µS/cm)	263.3 a ± 64.4	304.9 a ± 72.8	628.7 b ± 217.8	372.4 ± 200.9
	(199.7–396.3)	(224.3–376.3)	(209.7–855.7)	(199.7–855.7)
Color (Pfund scale)	51 a ± 17	56 a ± 15	96 b ± 7	65 ± 24
	(32–83)	(42–73)	(48–104)	(32–104)

EC: electrical conductivity. Different letters indicate significant differences according to Bonferroni test (*p* < 0.05) between the mean values by honey type. Total: all honey samples.

**Table 4:** Key volatile compounds of each studied honey type [12].

Botanical Origin	Volatile Compounds
*Atractylis* honey	Beta-eudesmol Alpha-bisabolol
*Eruca* honey	Dimethyl trisulfide Dimethyl tetrasulfide
*Retama* honey	Lilac aldehyde Lilac aldehyde D

## Data Availability

The original contributions presented in the study are included in the article, further inquiries can be directed to the corresponding author.
